# Evaluation of 3-Borono-l-Phenylalanine as a Water-Soluble Boron Neutron Capture Therapy Agent

**DOI:** 10.3390/pharmaceutics14051106

**Published:** 2022-05-22

**Authors:** Naoya Kondo, Fuko Hirano, Takashi Temma

**Affiliations:** Department of Biofunctional Analysis, Graduate School of Pharmaceutical Sciences, Osaka Medical and Pharmaceutical University, 4-20-1 Nasahara, Takatsuki 569-1094, Osaka, Japan; naoya.kondo@ompu.ac.jp (N.K.); ompu72121051@s.ompu.ac.jp (F.H.)

**Keywords:** boron neutron capture therapy, 4-borono-l-phenylalanine, drug discovery, solubility

## Abstract

Although 4-borono-l-phenylalanine (4-BPA) is currently the only marketed agent available for boron neutron capture therapy (BNCT), its low water solubility raises concerns. In this study, we synthesized 3-borono-l-phenylalanine (3-BPA), a positional isomer of 4-BPA, with improved water solubility. We further evaluated its physicochemical properties, tumor accumulation, and biodistribution. The water solubility of 3-BPA was 125 g/L, which is more than 100 times higher than that of 4-BPA. Due to the high water solubility, we prepared the administration solution of 3-BPA without a solubilizer sugar, which is inevitably added to 4-BPA preparation and has adverse effects. In in vitro and in vivo experiments, boron accumulation in cancers after administration was statistically equivalent in both sugar-complexed 3-BPA and 4-BPA. Furthermore, the biodistribution of 3-BPA was comparable with that of sugar-complexed 3-BPA. Since 3-BPA has high water solubility and tumor targetability equivalent to 4-BPA, 3-BPA can replace 4-BPA in future BNCT.

## 1. Introduction

Boron neutron capture therapy (BNCT) is a cancer treatment based on the nuclear reaction between boron (^10^B) atoms that are delivered into cancer cells and externally irradiated thermal neutrons. The alpha particles and Li nuclei produced in the nuclear reaction are high linear energy transfer radiation. They have a range of less than one cell’s length, so boronoagents that can selectively deliver ^10^B atoms into tumor cells allow for cancer-selective treatment [1,2,3]. To date, two boronoagents, 4-borono-l-phenylalanine (4-BPA) and sodium borocaptate (BSH), have been used clinically [4,5], and these studies demonstrated that 4-BPA was therapeutically more effective than BSH [6]. 4-BPA was discovered by Snyder et al. in 1958 [7], and Mishima et al. first succeeded in treating malignant melanoma with 4-BPA BNCT in 1989 [8]. Currently, 4-BPA is the most widely used boronoagent for BNCT [9,10,11]. In Japan, after positive results were shown in two clinical trials, 4-BPA was marketed under the name Borofalan (Steboronine^®^) in May 2020 for locally advanced or locally recurrent unresectable head and neck cancer [12]. As 4-BPA is taken up in cancer cells by L-type/large neutral amino acid transporter 1 (LAT1, SLC7A5) [13,14], a positron emission tomography (PET) scan with 2-[^18^F]fluoro-4-borono-l-phenylalanine, a LAT1 imaging probe, can noninvasively predict the applicability of 4-BPA BNCT [15]. Since LAT1 expression on plasma membrane has been reported to be cancer-specific [16], the agent with high LAT1 recognition ability can be expected to exhibit tumor-specific accumulation.

In clinical BNCT settings, the intratumor boron concentration must be maintained at a high level (>20 ppm) [17] for a 3 h treatment period because the therapeutic effect is directly proportional to the intratumor boron concentration. It is necessary to administer a large amount of boron; thus, high tumor targetability with high water solubility are prerequisites for a useful boronoagent. However, 4-BPA has extremely poor water solubility under neutral conditions (0.6–0.7 g/L), as mentioned in the Borofalan interview form; thus, it is solubilized by complex formation with sugars, such as fructose [18] and sorbitol [19], before administration. In particular, in the case of a patient with 60 kg body weight, 30 g 4-BPA, solubilized with 31.5 g d-sorbitol in 1000 mL administration solvent, is infused intravenously for treatment. Such a high load on the patient would have side effects. For example, hypoglycemia and liver and kidney failures could occur in patients with hereditary fructose intolerance due to fructose arising from d-sorbitol metabolism [20], and hematuria could also be a side effect due to the crystallization of 4-BPA in the urine resulting from its insolubility [12,21]. Thus, a critical drawback of 4-BPA is its low water solubility. However, it is quite challenging to develop boronoagents with high tumor targetability and high water solubility because the transmembrane transport system which is applicable for intracellular targeting of such a water-soluble boronoagent with an inferior passive intracellular penetration property to the high boron concentration levels required for BNCT is limited [3]. To date, no small molecular boronoagent other than 4-BPA and BSH has been tested in clinical trials [6].

**Figure 1:**
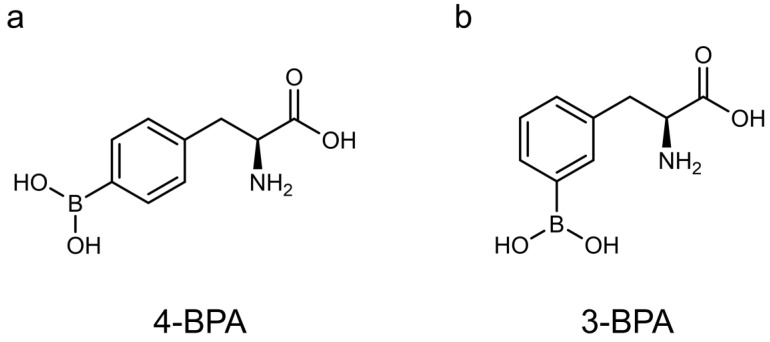
Chemical structure of 4-borono-l-phenylalanine (4-BPA, (**a**)) and 3-borono-l-phenylalanine (3-BPA, (**b**)).

We hypothesized that 3-borono-l-phenylalanine (3-BPA), a *meta* isomer of 4-BPA (Figure 1), has higher water solubility than 4-BPA, focusing on previous findings that *meta*-substituents show higher water solubility than *para*-substituents of the benzene ring due to asymmetric conformation and structural folding [22,23,24]. Furthermore, recent structure–activity relationship studies have shown that *meta*-substituted phenylalanine derivatives are transported more efficiently into cells by LAT1 than *para*-substituted derivatives [25,26]. Thus, 3-BPA would have improved water solubility and LAT1 recognition ability compared with 4-BPA. Although 3-BPA had been evaluated and concluded to be slightly inferior to 4-BPA in terms of accumulation in mouse melanoma in the 1990s [27,28], intraperitoneal administration had been adopted in these experiments targeting mouse melanoma regardless of LAT1 expression levels. Besides, the researchers added solubilizer sugar to the tested compounds, such as 2-, 3-, and 4-BPA, without estimating solubility in every experiment. Therefore, in this study, we carefully re-evaluated 3-BPA compared with 4-BPA in vitro and in vivo, focusing on water solubility and uptake through LAT1, and consequently found a significant superiority of 3-BPA as a promising lead compound of next-generation boronoagents in future BNCT.

## 2. Materials and Methods

### 2.1. General

All reagents were obtained commercially and used without further purification unless otherwise noted. 4-BPA (^10^B) was supplied by Stella Pharma Corp. (Osaka, Japan). Milli-Q water (Merck Millipore, Burlington, MA, USA) was used in all experiments.

### 2.2. Synthesis of 3-BPA

The synthesis of 3-BPA was performed according to previous reports [25,29,30,31]. The synthetic scheme is shown in Supplementary Figure S1, and the details are described in the Supplementary Methods. 3-BPA was purified and characterized using an RP-HPLC system equipped with a C18 reversed-phase column (COSMOSIL 5C18-AR-II 10 ID × 250 mm; Nacalai Tesque, Kyoto, Japan). The mobile phase was a mixture of water and acetonitrile (95:5, *v*/*v*) at a flow rate of 5.0 mL/min. This condition was also used for 4-BPA. The purified **3**-BPA was lyophilized to obtain fluffy white crystals and characterized by ESI-MS and ^1^H and ^13^C-NMR (Supplementary Figures S2 and S3).

### 2.3. Evaluation of the Physicochemical Properties of 3-BPA and 4-BPA

#### 2.3.1. Water Solubility

3-BPA or 4-BPA (40 mg) were suspended in 200 μL water and stirred at 25 °C for 24 h. After stirring, the solution was centrifuged at 5000 rpm for 10 min, and the amount of boron in the supernatant solution was measured by 8800 triple quadrupole ICP-MS (Agilent, Santa Clara, CA, USA). The experiment was repeated twice with n = 3.

#### 2.3.2. Log *p* Measurement

After dissolving 3-BPA or 4-BPA in water (0.2 mg/mL), 1-octanol was added to compose a water:1-octanol (1:1, *v*/*v*) mixture. The mixture was shaken for 5 min and then centrifuged at 5000 rpm for 10 min. The amount of boron was measured for each liquid phase using ICP-MS, and log *p* was calculated from the concentration ratio of each liquid phase. The experiment was repeated twice with n = 6.

### 2.4. In Vitro Experiments

#### 2.4.1. Cell Lines

B16F10 mouse melanoma cells (RCB2630) and T3M-4 human pancreatic adenocarcinoma cells (RCB1021) were provided by the RIKEN BRC (Ibaraki, Japan). U-87MG human glioblastoma cells were provided by Prof. Magata (Hamamatsu University School of Medicine, Hamamatsu, Japan), and A549 human lung carcinoma cells (CCL-185) were obtained from ATCC (Manassas, VA, USA). Cells were cultured in growth medium RPMI 1640 for B16F10 and T3M-4 and DMEM for A549 and U-87MG, containing 10% fetal bovine serum at 37 °C in a humidified atmosphere of 5% CO_2_.

#### 2.4.2. Western Blotting

Cultured cells lysed in Passive Lysis Buffer (Promega, Madison, WI, USA) were homogenized by sonication, centrifuged to remove debris, and subsequently diluted in 2-mercaptoethanol containing sample buffer (70 mM Tris, 1% SDS, 11% glycerol, 0.005% bromophenol blue). Samples (10 μL, 1.0 mg/mL) were then loaded, and Western blotting was performed using anti-LAT1 (KE026, 0.1 μg/mL) (Medicinal Chemistry Pharmaceutical, Sapporo, Japan) as a primary antibody and horseradish peroxidase-conjugated mAb as a secondary antibody (1:3000 dilution, HAF008, R&D Systems, Minneapolis, MN, USA). β-Actin levels were used to control protein loading in samples and were measured with anti-β-actin antibody (1:5000 dilution, NB600-505SS, Novus Biologicals, Littleton, CO, USA). Immunoreactive bands were visualized using Chemi-Lumi One L (Nacalai Tesque). The FastGene Bluestar prestained protein marker (NIPPON Genetics, Tokyo, Japan) was used as a molecular weight marker to estimate molecular weights. An Amersham Imager 600 (GE Healthcare Japan, Tokyo, Japan) was used to visualize bands. After ImageJ quantified the bands, the LAT1 expression levels among tumor cells were compared as the LAT1/β-actin ratio (n = 3).

#### 2.4.3. Cell Staining

The cells were fixed with 4% paraformaldehyde, permeabilized with 0.2% Triton X100, and incubated with Blocking One Histo (Nacalai Tesque) for 10 min, followed by incubation for 2 h at room temperature with an anti-LAT1 antibody (KE026, 0.1 μg/mL) as the primary antibody. The cells were then rinsed with PBS(-) and incubated for 30 min at room temperature with a CF555-labeled antibody (SAB4600068, 2 μg/mL, Sigma-Aldrich, St. Louis, MO, USA). For nuclear staining, the cells were incubated with Hoechst 33342 (5 μg/mL, Nacalai Tesque) for 10 min at room temperature. Fluorescence images were acquired with a BZ-X810 instrument (Keyence Japan Co., Osaka, Japan) and visualized by BZ-X800 Analyzer (Keyence Japan Co.).

#### 2.4.4. Cellular Uptake Study

Cells were cultured in six-well plates 2 days before the experiment. After medium removal, cells were washed twice with choline buffer (140 mM choline chloride, 2 mM potassium chloride, 1 mM magnesium chloride, 1 mM calcium chloride, 1 M Tris) [25] and preincubated with 900 µL of choline buffer containing/not containing 10 µM of the LAT1-specific inhibitor JPH203 [32,33] (Selleck Biotech, Tokyo, Japan) at 37 °C for 5 min. After incubation, 100 µL of 3-BPA or 4-BPA mixed with fructose (0.5 mg/mL, 0.11 *w*/*v*% Fru, pH 7.4) was added and incubated at 37 °C for 1, 5, and 30 min. After washing three times with choline buffer, 400 µL of 0.2 M NaOH was added to lyse the cells. After the amount of cell protein was measured using the BCA method (Thermo Fisher Scientific, Tokyo, Japan) and the mixture was ashed with nitric acid, the amount of boron was measured using ICP-MS. The accumulation rate was calculated as % dose/mg protein from the quantified dose of boron in added 3-BPA and 4-BPA, respectively (n = 3).

### 2.5. In Vivo Experiment

#### 2.5.1. Animal Preparation

Male BALB/c mice (4 weeks old, Japan SLC, Shizuoka, Japan, n = 35) and BALB/c nu-nu mice (4 weeks old, Japan SLC, n = 18) were housed under a 12 h light/12 h dark cycle and given free access to food and water. Animal experiments were conducted according to the institutional guidelines for animal experiments. The study protocol was approved by the institutional Experimental Animal Committee (Permission Number: 19-76 and 20-76). Tumor-bearing mice were prepared by subcutaneous inoculation of B16F10 cells (5 × 10^5^ cells/mouse) suspended in PBS (-) solution (100 μL) into the right hind legs of BALB/c mice or T3M-4 cells (2.5 × 10^6^ cells/mouse) suspended in PBS (-):Matrigel = 1:1 solution (100 μL) into the right hind legs of BALB/c nu-nu mice. The treatments were performed under 3–5% isoflurane anesthesia to reduce pain in the mice. Animals with a tumor of approximately 10 mm in diameter at 5 weeks after inoculation of cells were assigned for in vivo biodistribution studies so that tumor size was not biased between groups. Mice without viable T3M-4 tumors (n = 5) were euthanized by isoflurane inhalation overdose.

#### 2.5.2. In Vivo Biodistribution Study

B16F10-bearing mice were intravenously injected with 3-BPA or 4-BPA mixed with fructose (3-BPA-Fru, 4-BPA-Fru, 1 mg/100 μL PBS (-), 2.2 *w*/*v*% Fru, pH 7.4), and sacrificed 10, 30, 60, or 120 min after administration. Samples of the plasma and the tissues of interest were excised, weighed, and ashed by nitric acid, followed by ICP-MS measurements of the boron amount. The accumulation rate was calculated as the % injected dose/g (%ID/g) of the quantified dose of boron in the administrated solution.

LAT1-positive T3M-4 xenograft mice were intravenously injected with 3-BPA-Fru, 4-BPA-Fru (1 mg/100 μL PBS (-), 2.2 *w*/*v*% Fru, pH 7.4), or fructose-free 3-BPA (1 mg/100 μL PBS (-)) and sacrificed 60 min later, followed by boron amount measurement in the same manner. The details of the preparation of 3-BPA-Fru and 4-BPA-Fru and measurement of ^11^B-NMR (DD2 NMR Spectrometer, Agilent, Santa Clara, CA, USA, 600 MHz) to confirm the formation of the complex with fructose are described in Supplementary Methods.

### 2.6. Statistics

Data are presented as means ± standard deviations. Statistical analyses were performed using Tukey’s multiple comparison tests or an unpaired *t*-test with GraphPad Prism 8. Differences at the 95% confidence level (*p* < 0.05) were considered significant unless otherwise noted.

## 3. Results

### 3.1. Properties of the 3-BPA and 4-BPA

The physicochemical properties of 3-BPA and 4-BPA are summarized in Table 1.

Notably, the solubility of 3-BPA in water was 125 ± 12 g/L, which is more than 100 times higher than that of 4-BPA (0.72 ± 0.13 g/L) (n = 4, each). 3-BPA has a significantly higher log *p* value (n = 6) and a longer RP-HPLC retention time than 4-BPA.

### 3.2. Cellular Uptake Study

**Figure 2:**
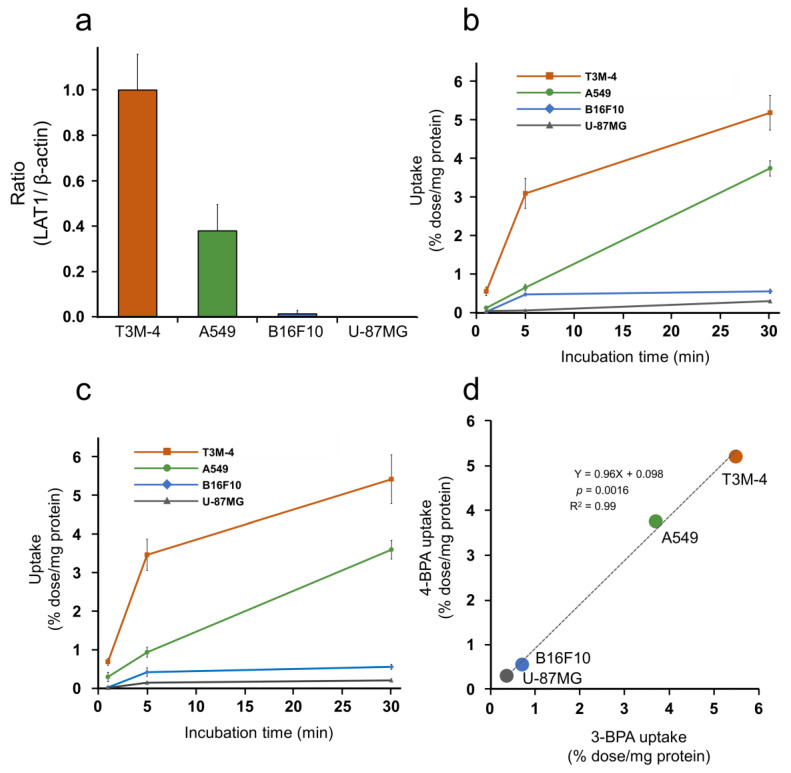
In vitro cell uptake of 3-BPA and 4-BPA in cancer cells with various LAT1-expression. (**a**) The LAT1/β-actin ratio of each cell calculated from the expression levels of LAT1 and β-actin obtained by Western blotting and standardized to a value of 1 for T3M-4. (**b**,**c**) LAT1-specific accumulation (the difference in accumulation between the inhibitor group and the noninhibitor group) in T3M-4, A549, B16F10, and U-87MG cells after 1, 5, and 30 min incubation with 3-BPA (**b**) and 4-BPA (**c**). (**d**) The relationship between the uptake (% dose/mg protein) of 3-BPA and 4-BPA at 30 min incubation with a simple linear regression. Statistical analyses of LAT1-specific accumulation of 3-BPA (**b**) and 4-BPA (**c**) were shown in Supplementary Tables S3 and S4, respectively.

Representative bands from Western blotting of T3M-4, A549, U-87MG, and B16F10 cells and the intensity ratio to the β-actin band are shown in Supplementary Figure S4 and Figure 2a, respectively. Representative images of cellular immunofluorescence staining are shown in Supplementary Figure S5. These results indicated that the LAT1 expression levels were decreased in each cell type in the following order: T3M-4, A549, B16F10, and U-87MG cells. Boron uptakes in each cell line after 3-BPA and 4-BPA additions are summarized in Supplementary Tables S1 and S2, showing significant inhibition of boron uptake by LAT1 inhibitor treatment in all cell groups. The LAT1-specific boron uptake calculated from the difference in accumulations between the inhibitor and noninhibitor groups is plotted with time in Figure 2b,c. LAT1-specific boron uptake after the addition of 3-BPA and 4-BPA was correlated with the expression level of LAT1 (Figure 2a), which was confirmed by statistical analysis (Supplementary Tables S3 and S4). Further, simple linear regression (Figure 2d) and Tukey’s multiple comparison tests (Supplementary Table S5) showed substantial equivalence between the LAT1-specific boron uptakes of 3-BPA and 4-BPA in vitro.

### 3.3. Biodistribution Study

**Figure 3:**
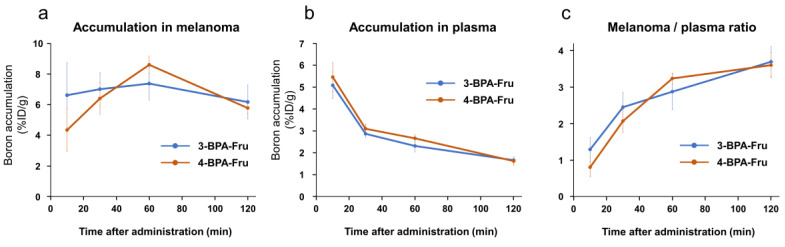
Boron accumulation in B16F10 melanoma (**a**) and plasma (**b**), and melanoma to plasma ratio of boron accumulation (**c**) of 3-BPA-Fru and 4-BPA-Fru at 10, 30, 60, and 120 min after administration.

^11^B-NMR analysis of 3-BPA-Fru, 4-BPA-Fru, and 3-BPA showed chemical shifts of ^11^B atoms in 9.9, 10.1, and 30.6 ppm, respectively (Supplementary Figures S6–S8). It indicated a change in electron density around ^11^B atoms due to fructose complex formation in 3-BPA-Fru and 4-BPA-Fru solutions. Biodistributions after intravenous administration of 3-BPA-Fru and 4-BPA-Fru to B16F10 melanoma-bearing mice are summarized in Table 2 and Table 3, and significant differences between 3-BPA-Fru and 4-BPA-Fru in boron accumulations are summarized in Supplementary Table S6. Both exhibited high accumulation in the pancreas, which is a high LAT1-expressing organ [34], whereas 3-BPA-Fru showed a significantly higher boron level in the kidneys than 4-BPA-Fru. The boron levels in the melanoma and plasma and the melanoma to plasma ratios were not significantly different between 3-BPA-Fru and 4-BPA-Fru groups over time (Figure 3).

**Figure 4:**
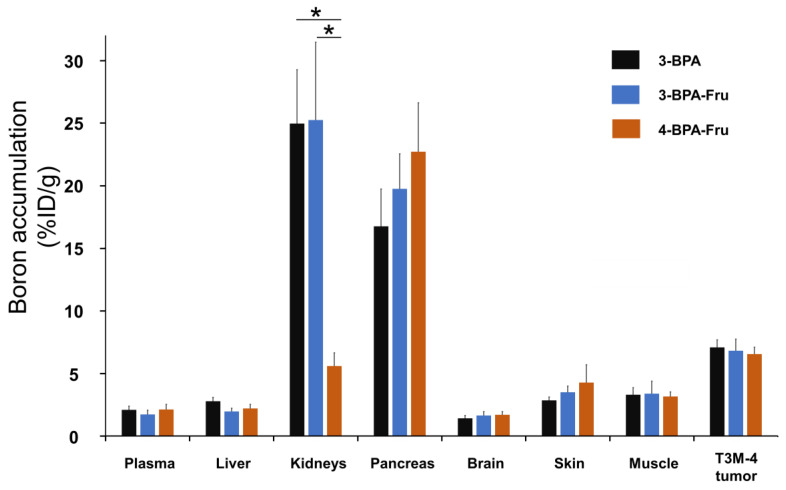
Boron accumulation in T3M-4 tumor, plasma, liver, kidneys, and the tissues surrounding the tumor at 60 min after administration of 3-BPA, 3-BPA-Fru, and 4-BPA-Fru into BALB/c nu-nu mice. * *p* < 0.001 by Tukey’s multiple comparison tests.

Biodistributions 60 min after intravenous administration of 3-BPA-Fru, 4-BPA-Fru, and 3-BPA to LAT1-positive T3M-4-bearing mice are summarized in Figure 4 and Supplementary Table S7. Similar to the above results, there were no significant differences among groups in boron accumulation levels in the organs, except for the kidneys. Note that 3-BPA and 3-BPA-Fru showed similar boron levels in all evaluated organs, indicating no additive function of fructose on drug pharmacokinetics.

## 4. Discussion

As 4-BPA has a low water solubility that can cause side effects in patients with BNCT, this study aimed to develop an alternative boronoagent and re-evaluated 3-BPA compared with 4-BPA in water solubility and tumor targetability through LAT1. We found that 3-BPA is highly soluble in water, more than 100 times higher than 4-BPA. As 3-BPA and 4-BPA have similar acid dissociation constants (3-BPA: pKa_1_ = 2.26, pKa_2_ = 8.46, pKa_3_ = 9.95; 4-BPA: pKa_1_ = 2.35, pKa_2_ = 8.45, pKa_3_ = 9.67) [35], both would exist in similar charge states in the neutral solution. The log *p* values and retention times in RP-HPLC suggest a slightly higher tendency of 3-BPA toward the organic layer than 4-BPA. Although this would appear contradictory to the solubility result, a relationship between tyrosine and phenylalanine is similar to this case. Tyrosine shows a lower log *p* value but a lower water solubility than phenylalanine (log *p* value, −2.26 [36] vs. −1.38 [36]; water solubility, 0.47 g/L [37] vs. 12.0 g/L [38]), indicating that the hydroxyl group present at the *para*-position of phenylalanine contributes to a decrease in water solubility. Similarly, 4-BPA shows a lower log *p* value and water solubility than phenylalanine. In addition, to clarify the substituent position effect, we measured and compared the water solubility of dl-*meta*-tyrosine and dl-tyrosine (Supplementary Table S8). The results showed more than 25 times higher water solubility of dl-*meta*-tyrosine than dl-tyrosine, confirming that introducing a substituent at *meta*-position improves water solubility. The improved water solubility of 3-BPA may be attributed to the difference in thermodynamically stable conformations from 4-BPA, leading to increased intermolecular interactions with the surrounding water molecules and decreased π–π interaction within boron agents, but further studies are required to understand the detailed mechanism.

In in vitro experiments, we initially used B16F10 cells as a positive control following past BNCT studies [28,39,40]. Surprisingly, we still found low LAT1 expression and boron accumulation after 3-BPA and 4-BPA addition compared with other LAT1-expressing cell groups. In the past, research was focused on melanoma, which was the primary target disease of BNCT at that time, and no further evaluation was conducted when slightly less accumulation of 3-BPA was observed in melanoma cells [27]. Nevertheless, in the present experiments using high LAT1 expressing cells, we have succeeded in demonstrating a LAT1-dependent accumulation profile of 3-BPA statistically equivalent to that of 4-BPA, which supports our decision to re-evaluate.

In clinical BNCT, the boron accumulations in the tumor and surrounding normal tissues are both considered because neutron exposure to the surrounding tissues is inevitable during neutron irradiation and can have side effects [41,42]. This study evaluated the amount of boron accumulation in the brain, skin, and muscle as peripheral tissues in addition to the tumor. 3-BPA-Fru and 4-BPA-Fru showed equivalent biodistribution profiles in the tumor and peripheral tissues in conventional melanoma mice, suggesting similar effectiveness of 3-BPA-Fru as a boronoagent in BNCT. A significant boron accumulation observed in the kidneys after 3-BPA-Fru injection could come from a possible recognition difference in renal transporter systems, but it could not cause crystallization and hematuria due to the improved solubility. The mechanism of the accumulation difference in the kidneys remains unclear, and further studies are needed to determine the associated effects. Interestingly, in vitro and in vivo tumor boron accumulation after 3-BPA-Fru administration was perfectly similar to the level obtained by 4-BPA-Fru, unlike our prediction based on the previous reports showing the superiority of *meta*-substituted phenylalanine in LAT1 recognition [25,26]. Since these studies did not include boronic acid substitution [25,26], further research on the structure–activity relationship of compounds with boronic acid substitution using 3-BPA as a lead compound will contribute to BNCT agent development.

We evaluated the biodistribution of 3-BPA without fructose solubilization due to highly improved solubility. The boron concentration in the administration solution was more than 10 times higher than the solubility of 4-BPA. The biodistribution of 3-BPA was comparable to that of 3-BPA-Fru, indicating that there was no need for fructose addition in the preparation. In a tentative estimate, for a patient with 60 kg of body weight, 250 mL or less of 3-BPA solution is required without solubilizer addition, in contrast to the case of 4-BPA, which includes 31.5 g d-sorbitol in 1000 mL solution. Details of Borofalan marketization that fructose was changed to d-sorbitol due to unsuitability for long-term storage by the Maillard reaction [43], may support that 3-BPA prepared without solubilizer addition has advantages in terms of quality control. In the clinical settings, 4-BPA would cause crystalluria and hematuria because the solubility is lower than the possible urinary concentration level excreted from the body [44], but the solubility of 3-BPA is much higher than the estimated urinary concentration level, thus eliminating such related side effects. Next, 3-[^10^B]BPA synthesized by an optimized synthetic scheme needs to be assessed using the intravenous infusion method before its application in clinical settings. The tumor targetability of 3-BPA should also be evaluated at clinical doses in the future, with reference to the report that transporters other than LAT1 contribute to tumor uptake at clinical doses of 4-BPA [14]. As 4-BPA was initially developed to target melanoma [8,45], 4-BPA was designed according to the structure of tyrosine, the precursor of melanin. Since then, 4-BPA has been promoted for clinical use, and several agents based on the 4-BPA structure have been developed [46,47]. As the *meta*-substituent improved water solubility and sustained tumor targetability, 3-BPA can replace 4-BPA as the lead compound for future drug design of boronoagents.

In summary, although future verification for clinical use is needed, this study demonstrated that 3-BPA is highly water-soluble, enables solubilizer elimination unlike 4-BPA, and has a biodistribution property comparable to 4-BPA. Therefore, 3-BPA is a promising BNCT agent that surpasses the currently marketed 4-BPA.

## Figures and Tables

**Table 1:** Physicochemical properties of 3-BPA and 4-BPA.

	3-BPA	4-BPA
Solubility (in water, 25 °C, n = 6)	* 125 ± 12 (g/L)	0.72 ± 0.13 (g/L)
Log *p* (n = 12)	^†^ −1.59 ± 0.03	−1.80 ± 0.04
RP-HPLC Retention time	4.5 (min)	3.7 (min)

* *p* < 0.0001 vs. 4-BPA by unpaired *t*-test. *t* = 24.24, df = 10. ^†^ *p* < 0.0001 vs. 4-BPA by unpaired *t*-test. *t* = 15.41, df = 22.

**Table 2:** Biodistribution of boron concentration after administration of 3-BPA-Fru in melanoma-bearing mice (%ID/g, n = 19).

	Time after Administration (min)			
	10 (n = 4)	30 (n = 4)	60 (n = 7)	120 (n = 4)
Plasma	5.1 ± 0.6	2.9 ± 0.2	2.3 ± 0.3	1.7 ± 0.1
Liver	8.4 ± 0.6	4.6 ± 0.4	3.5 ± 0.9	2.6 ± 0.3
Kidneys	58.3 ± 11.3	53.6 ± 7.1	36.4 ± 8.4	23.0 ± 1.9
Pancreas	52.5 ± 4.9	50.2 ± 4.5	33.1 ± 8.5	34.4 ± 2.9
Brain	2.6 ± 0.3	3.0 ± 0.1	3.0 ± 0.9	3.4 ± 0.5
Skin	5.3 ± 0.4	4.2 ± 0.5	3.4 ± 0.5	2.7 ± 0.6
Muscle	4.5 ± 0.8	5.1 ± 0.3	4.6 ± 0.5	4.4 ± 0.5
B16F10 melanoma	6.6 ± 2.1	7.0 ± 1.1	7.4 ± 1.0	6.2 ± 1.1
Tumor/Plasma	1.3 ± 0.3	2.4 ± 0.4	2.9 ± 0.5	3.7 ± 0.4

**Table 3:** Biodistribution of boron concentration after administration of 4-BPA-Fru in melanoma-bearing mice (%ID/g, n = 16).

	Time after Administration (min)			
	10 (n = 4)	30 (n = 4)	60 (n = 4)	120 (n = 4)
Plasma	5.5 ± 0.7	3.1 ± 0.2	2.7 ± 0.2	1.6 ± 0.2
Liver	7.4 ± 0.7	4.2 ± 0.2	2.9 ± 0.3	2.0 ± 0.1
Kidneys	16.9 ± 2.5	10.8 ± 1.6	8.4 ± 3.7	4.7 ± 0.6
Pancreas	44.1 ± 7.0	44.8 ± 5.7	33.9 ± 6.5	12.9 ± 1.4
Brain	1.3 ± 0.1	2.5 ± 0.2	3.0 ± 0.3	2.2 ± 0.2
Skin	5.2 ± 0.7	4.4 ± 0.5	5.0 ± 0.9	2.5 ± 0.3
Muscle	3.1 ± 0.7	4.2 ± 0.4	4.4 ± 0.3	2.8 ± 0.3
B16F10 melanoma	4.3 ± 1.4	6.4 ± 1.0	8.6 ± 0.6	5.8 ± 0.4
Tumor/Plasma	0.8 ± 0.3	2.1 ± 0.3	3.2 ± 0.1	3.6 ± 0.4

## Data Availability

The data supporting the results and findings of this study are available within the paper and the Supplementary Information files. Additional raw data are available from the corresponding author upon request.

## Supplementary Materials

The following supporting information can be downloaded at: https://www.mdpi.com/article/10.3390/pharmaceutics14051106/s1, Figure S1: Synthetic scheme of 3-BPA, Figure S2: ^1^H-NMR spectrum of 3-BPA, Figure S3: ^13^C-NMR spectrum of 3-BPA, Figure S4: Representative western blot bands (LAT1 and β-actin) of cell lysate, Figure S5: Representative images of fluorescence immunostaining of LAT1 and nuclear staining in cells, Figure S6: ^11^B-NMR spectrum of 3-BPA in PBS(-), Figure S7: ^11^B-NMR spectrum of 3-BPA-Fru in PBS(-), Figure S8: ^11^B-NMR spectrum of 4-BPA-Fru in PBS(-); Table S1: Boron uptake in cancer cells after incubation with 3-BPA, Table S2: Boron uptake in cancer cells after incubation with 4-BPA, Table S3: Statistical analysis of specific uptake between each cell after addition of 3-BPA, Table S4: Statistical analysis of specific uptake between each cell after addition of 4-BPA, Table S5: Statistical analysis of specific uptake between 3-BPA and 4-BPA in each cell, Table S6: Significant difference between 3-BPA-Fru and 4-BPA-Fru in boron accumulation in each tissue after administration into melanoma-bearing mice, Table S7: Biodistribution of boron concentration at 60 min after injection of 3-BPA, 3-BPA-Fru, and 4-BPA-Fru in T3M-4 tumor-bearing mice, Table S8: Water solubility of phenylalanine, tyrosine, and meta-tyrosine; Supplementary Methods.
