# Supplementary material for: Evaluation of 3-Borono-l-Phenylalanine as a Water-Soluble Boron Neutron Capture Therapy Agent
Source: Pharmaceutics. 2022 May 22;14(5):1106. doi: 10.3390/pharmaceutics14051106 (PMC9143228; doi:10.3390/pharmaceutics14051106)
Supplement: Supplementary file 1 [file pharmaceutics-14-01106-s001.zip › pharmaceutics-1731181-supplementary.pdf]

Supplementary Information for:

Evaluation of 3-borono-*L*-phenylalanine as a water-soluble boron neutron capture therapy agent.

Supplementary Figures

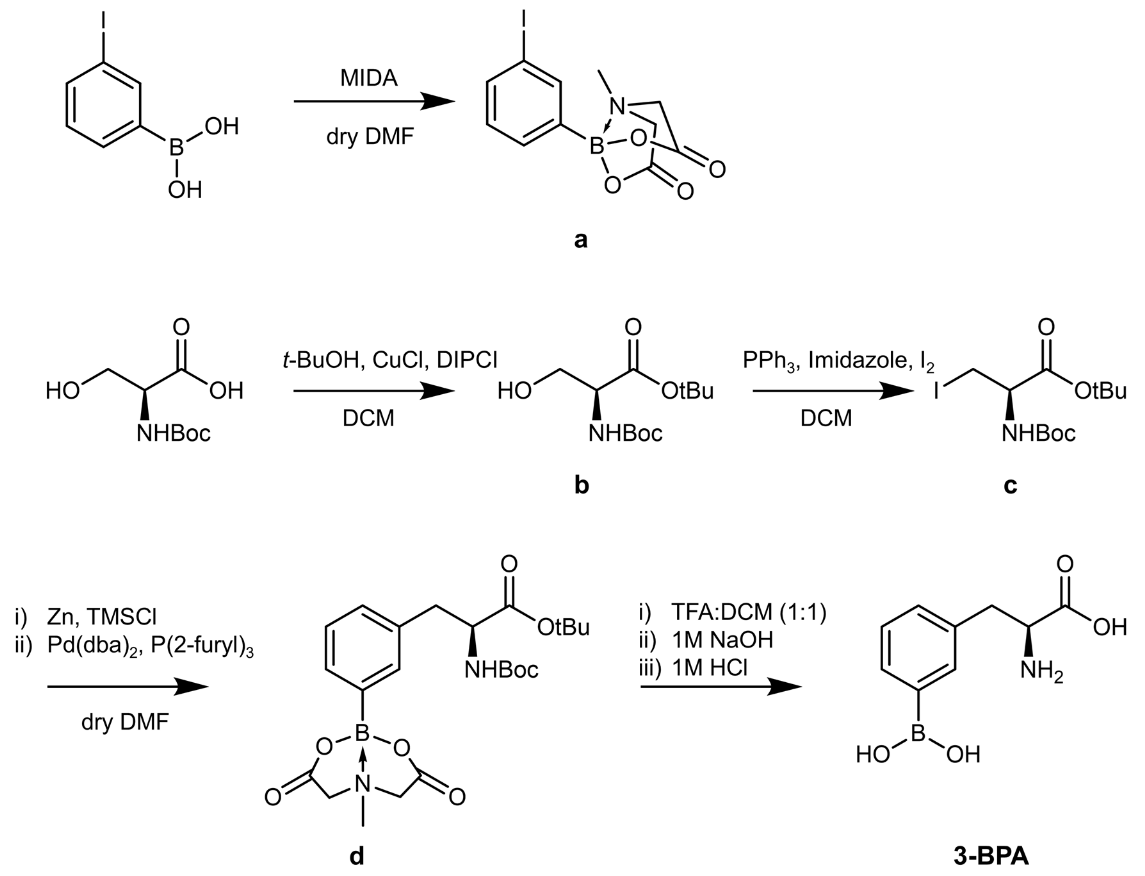

Supplementary Figure S1 Synthetic scheme of 3-BPA

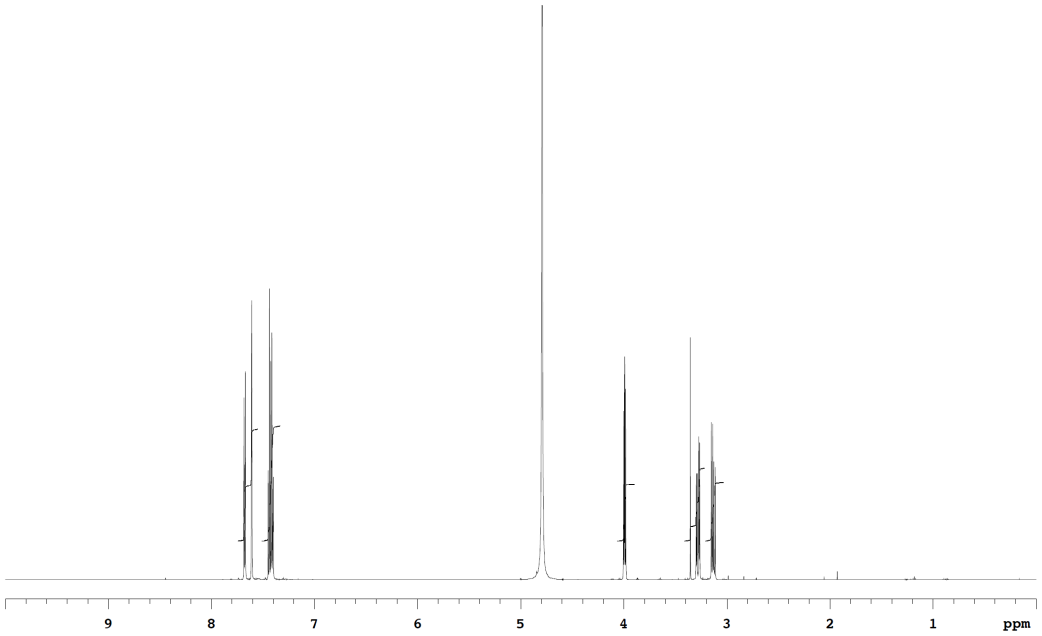

Supplementary Figure S2 ^{1}H-NMR spectrum of 3-BPA.

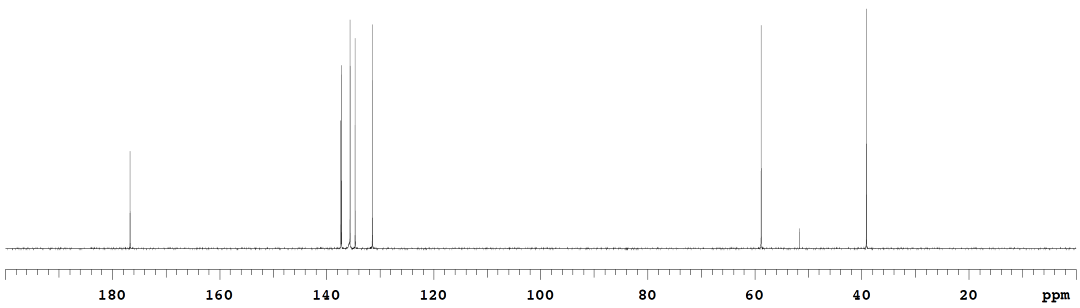

Supplementary Figure S3 ^{13}C-NMR spectrum of 3-BPA.

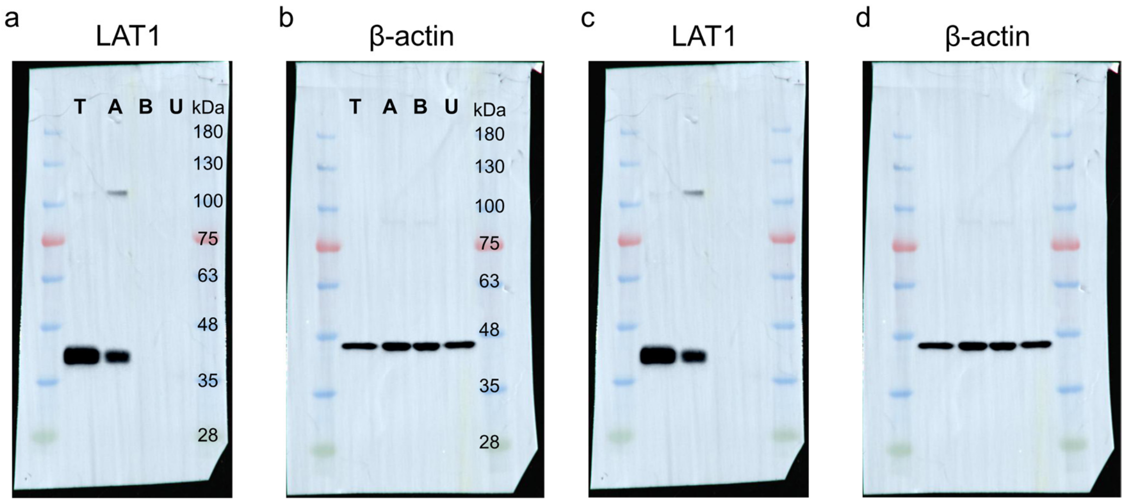

Supplementary Figure S4 Representative western blot bands: LAT1(39 kDa) **(a)** and β-actin (45 kDa) **(b)** in lysate of in T3M-4 (T), A549 (A), B16F10 (B), and U-87MG (U) cells with the molecular weight markers (FastGene Bluestar prestained protein marker). The corresponding molecular weights are shown with reference to the instructions for the molecular weight markers. **c** and **d** represent the membranes of a and b, respectively, which have not been modified at all.

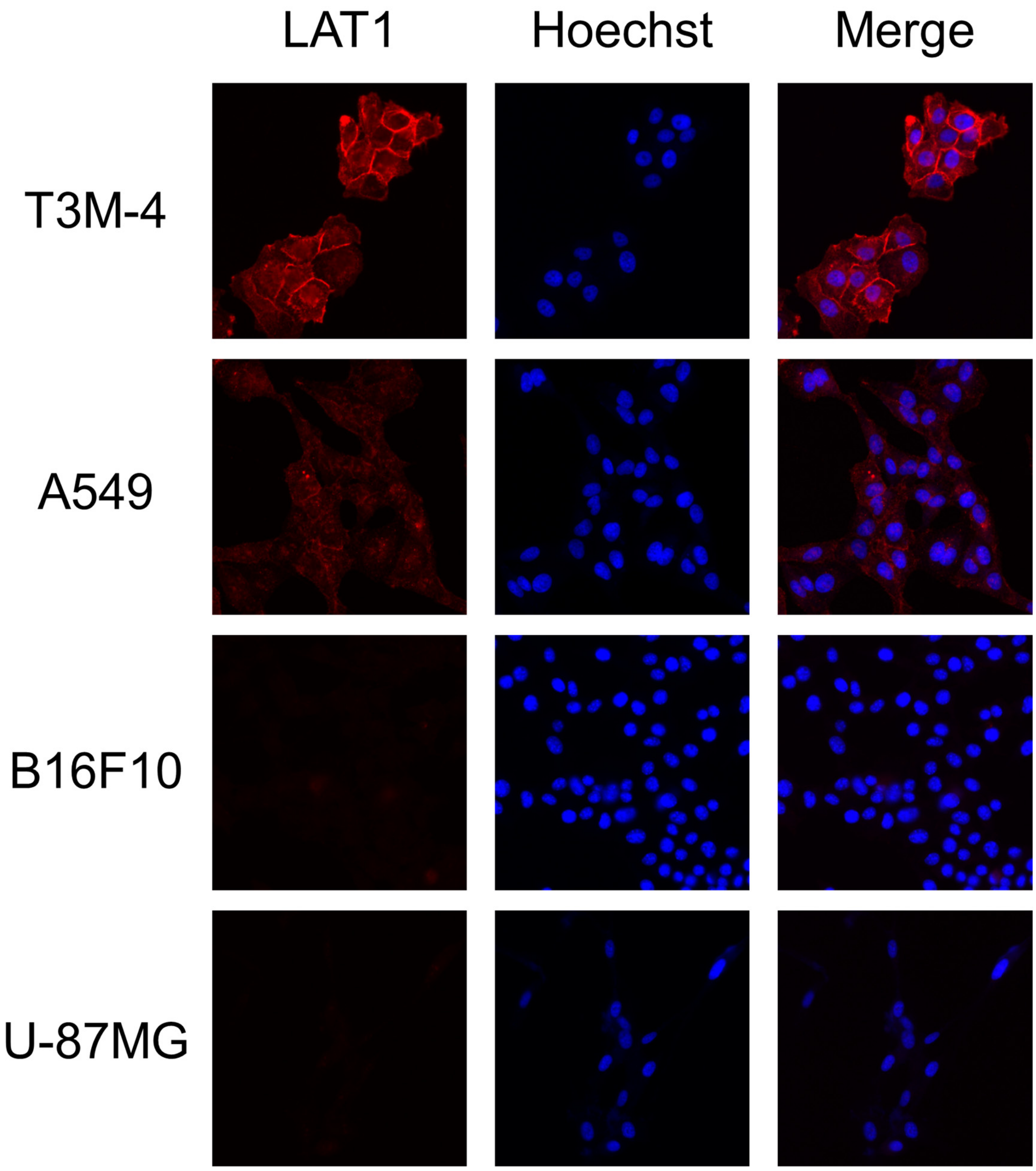

Supplementary Figure S5 Representative images of fluorescence immunostaining of LAT1 and nuclear staining using Hoechst 33342 in T3M-4, A549, B16F10, and U-87MG cells. Merge represents merging of images for anti-LAT1 and Hoechst 33342 staining.

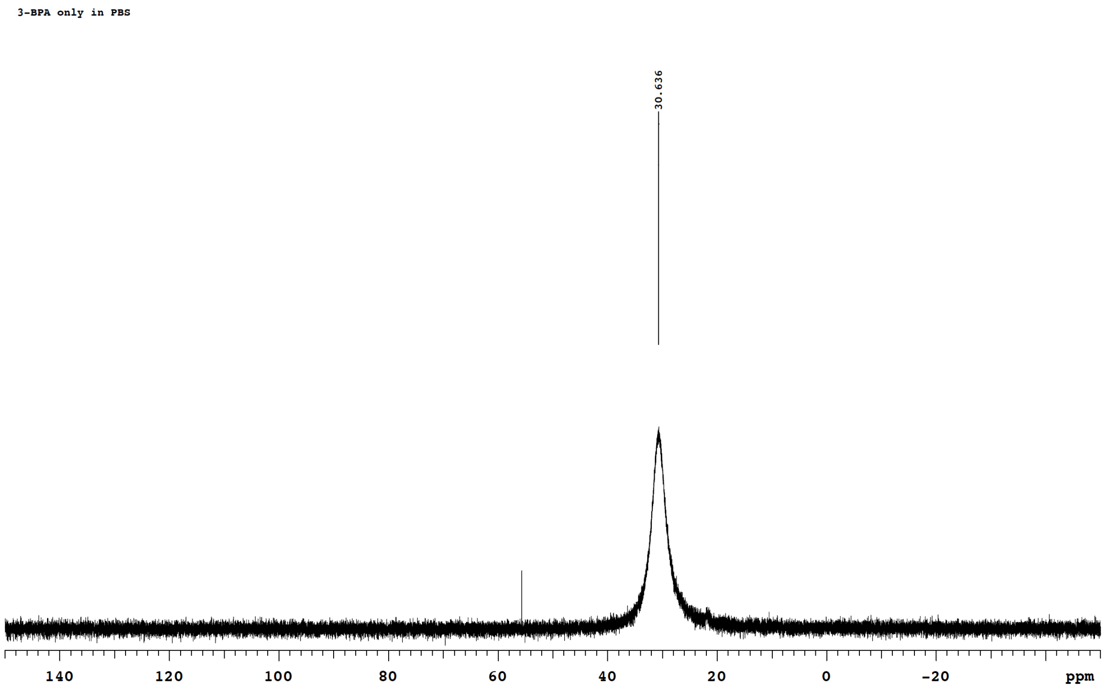

Supplementary Figure S6 ^{11}B-NMR spectrum of 3-BPA in PBS(-).

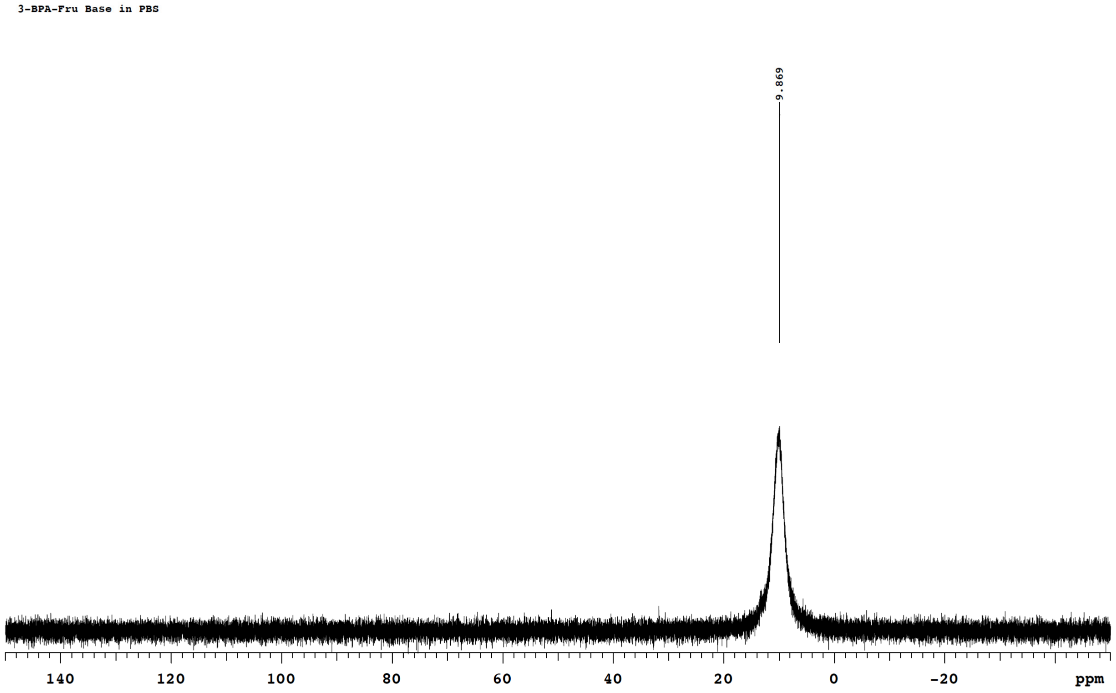

Supplementary Figure S7 ^{11}B-NMR spectrum of 3-BPA-Fru in PBS(-)

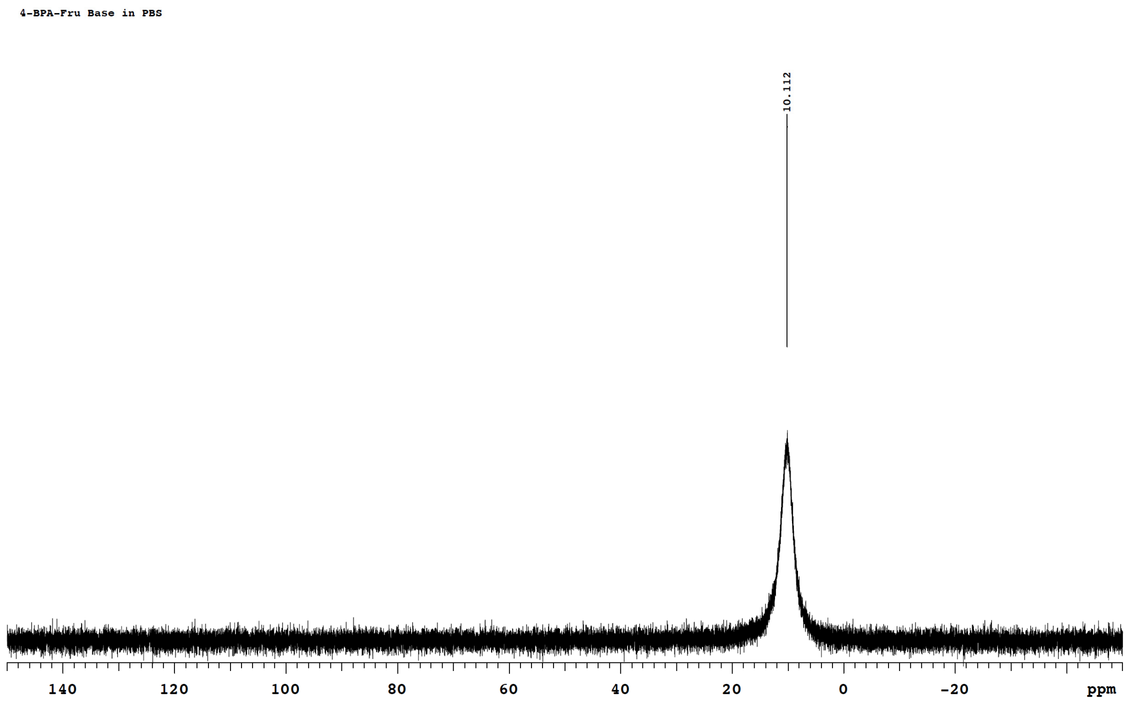

Supplementary Figure S8 ^{11}B-NMR spectrum of 4-BPA-Fru in PBS(-)

Supplementary Table S1

Boron uptake in cancer cells after incubation with 3-BPA (% dose/mg protein)

		1 min	5 min	30 min
T3M-4	non-inhibitor	0.78 ± 0.09	3.55 ± 0.40	5.62 ± 0.63
	inhibitor	***0.09 ± 0.08	***0.09 ± 0.07	***0.21 ± 0.06
	specific	0.69 ± 0.09	3.55 ± 0.40	5.41 ± 0.63
A549	non-inhibitor	0.48 ± 0.12	1.22 ± 0.13	4.02 ± 0.24
	inhibitor	*0.18 ± 0.03	***0.29 ± 0.02	***0.42 ± 0.01
	specific	0.30 ± 0.12	0.94 ± 0.40	3.60 ± 0.24
B16F10	non-inhibitor	0.23 ± 0.03	0.59 ± 0.11	0.80 ± 0.07
	inhibitor	0.21 ± 0.06	**0.17 ± 0.11	***0.24 ± 0.01
	specific	0.03 ± 0.03	0.42 ± 0.11	0.56 ± 0.07
U-87MG	non-inhibitor	0.33 ± 0.04	0.28 ± 0.04	0.45 ± 0.05
	inhibitor	0.31 ± 0.05	*0.14 ± 0.05	**0.24 ± 0.04
	specific	0.02 ± 0.04	0.15 ± 0.04	0.21 ± 0.05

* $p < 0.05$, ** $p < 0.01$, *** $p < 0.001$ v.s. Corresponding non-inhibitor group by unpaired t test.

Supplementary Table S2

Boron uptake in cancer cells after incubation with 4-BPA (% dose/mg protein)

		1 min	5 min	30 min
T3M-4	non-inhibitor	0.82 ± 0.11	3.28 ± 0.39	5.40 ± 0.45
	inhibitor	**0.26 ± 0.16	***0.18 ± 0.02	***0.20 ± 0.01
	specific	0.56 ± 0.11	3.10 ± 0.39	5.19 ± 0.45
A549	non-inhibitor	0.14 ± 0.04	0.66 ± 0.08	3.92 ± 0.20
	inhibitor	**0.01 ± 0.01	***0.00 ± 0.01	***0.17 ± 0.09
	specific	0.13 ± 0.04	0.66 ± 0.08	3.75 ± 0.20
B16F10	non-inhibitor	0.03 ± 0.01	0.62 ± 0.01	0.57 ± 0.06
	inhibitor	**0.00 ± 0.01	***0.14 ± 0.03	***0.01 ± 0.01
	specific	0.03 ± 0.01	0.48 ± 0.01	0.56 ± 0.06
U-87MG	non-inhibitor	0.13 ± 0.02	0.20 ± 0.02	0.48 ± 0.03
	inhibitor	*0.08 ± 0.01	*0.14 ± 0.02	***0.17 ± 0.01
	specific	0.05 ± 0.02	0.06 ± 0.02	0.31 ± 0.03

* $p < 0.05$, ** $p < 0.01$, *** $p < 0.001$ v.s. Corresponding non-inhibitor group by unpaired t test.

Supplementary Table S3

Statistical analysis of specific uptake between each cell after addition of 3-BPA

		1 min		5 min		30 min	
3-BPA	T vs. A	***	0.0001	****	<0.0001	****	<0.0001
	T vs. B	****	<0.0001	****	<0.0001	****	<0.0001
	T vs. U	****	<0.0001	****	<0.0001	****	<0.0001
	A vs. B	**	0.0048	n.s.	0.116	****	<0.0001
	A vs. U	**	0.0037	**	0.0056	****	<0.0001
	B vs. U	n.s.	>0.9999	n.s.	0.7453	n.s.	0.825

** $p < 0.01$, *** $p < 0.001$, **** $p < 0.001$ by Tukey's multiple comparisons test.
n.s.; not significant, T;T3M-4: A;A549: B;B16F10: U;U-87MG.

Supplementary Table S4

Statistical analysis of specific uptake between each cell after addition of 4-BPA

		1 min		5 min		30 min	
4-BPA	T vs. A	****	<0.0001	****	<0.0001	***	0.0004
	T vs. B	****	<0.0001	****	<0.0001	****	<0.0001
	T vs. U	****	<0.0001	****	<0.0001	****	<0.0001
	A vs. B	n.s.	0.6931	n.s.	0.9586	****	<0.0001
	A vs. U	n.s.	0.8681	*	0.0495	****	<0.0001
	B vs. U	n.s.	>0.9999	n.s.	0.2963	n.s.	0.9624

* $p < 0.05$, *** $p < 0.001$, **** $p < 0.001$ by Tukey's multiple comparisons test.

n.s.; not significant, T;T3M-4: A;A549: B;B16F10: U;U-87MG.

Supplementary Table S5

Statistical analysis of specific uptake between 3-BPA and 4-BPA in each cell

	1 min		5 min		30 min	
T3M-4	n.s.	0.3823	n.s.	0.4601	n.s.	0.9803
A549	n.s.	0.1309	n.s.	0.7283	n.s.	0.9976
B16F10	n.s.	>0.9999	n.s.	>0.9999	n.s.	>0.9999
U-87MG	n.s.	0.9992	n.s.	0.9995	n.s.	0.9999

n.s.; not significant by Tukey's multiple comparisons test.

Supplementary Table S6

Significant difference between 3-BPA-Fru and 4-BPA-Fru in boron accumulation in each tissue after administration into melanoma-bearing mice

	Time after administration (min)							
	10		30		60		120	
Plasma	n.s.	0.4364 (0.8336)	n.s.	0.1447 (1.676)	n.s.	0.2807 (1.148)	n.s.	0.7158 (0.3817)
Liver	n.s.	0.0691 (2.211)	n.s.	0.1549 (1.627)	n.s.	0.2327 (1.280)	*	0.0137 (3.448)
Kidneys	***	0.0004 (7.175)	****	<0.0001 (11.74)	***	0.0001 (6.266)	****	<0.0001 (18.03)
Pancreas	n.s.	0.0974 (1.962)	n.s.	0.1867 (1.490)	n.s.	0.8750 (0.1619)	****	<0.0001 (13.25)
Brain	***	0.0001 (8.895)	*	0.0127 (3.508)	n.s.	0.9623 (0.0486)	**	0.0036 (4.624)
Skin	n.s.	0.6871 (0.4228)	n.s.	0.7062 (0.3955)	**	0.0045 (3.756)	n.s.	0.7175 (0.3793)
Muscle	*	0.0296 (2.840)	*	0.0141 (3.423)	n.s.	0.3898 (0.9034)	**	0.0018 (5.339)
B16F10 melanoma	n.s.	0.1216 (1.802)	n.s.	0.4488 (0.8101)	n.s.	0.0655 (2.096)	n.s.	0.5229 (0.6782)
Tumor/Plasma	n.s.	0.0599 (2.314)	n.s.	0.1886 (1.483)	n.s.	0.9179 (0.106)	n.s.	0.7374 (0.3512)

P-values in the top row and t-values in the bottom row shown in parentheses.

* $p < 0.05$, ** $p < 0.01$, *** $p < 0.001$, **** $p < 0.001$ by unpaired t test.

n.s.; not significant, degrees of freedom(df);6 for 10, 30 and 120 min, df=9 for 60 min

Supplementary Table S7

Biodistribution of boron concentration (%ID/g) at 60 min after injection of 3-BPA, 3-BPA-Fru, and 4-BPA-Fru in T3M-4 tumor-bearing mice

	3-BPA (n = 4)	3-BPA-Fru (n = 5)	4-BPA-Fru (n = 4)
Plasma	2.1 ± 0.3	1.7 ± 0.3	2.1 ± 0.3
Liver	2.8 ± 0.3	2.1 ± 0.5	2.1 ± 0.1
Kidney	25.0 ± 4.3	24.5 ± 4.1	***6.3 ± 0.9
Pancreas	16.8 ± 0.6	19.2 ± 2.5	18.9 ± 6.5
brain	1.4 ± 0.2	1.5 ± 0.3	1.7 ± 0.2
Skin	2.9 ± 0.3	3.2 ± 0.7	3.2 ± 1.3
Muscle	3.3 ± 0.6	3.7 ± 1.0	3.1 ± 0.5
T3M-4 Tumor	7.1 ± 0.6	6.1 ± 0.5	6.1 ± 1.1
T3M-4/Plasma	3.4 ± 0.5	3.7 ± 0.7	2.8 ± 0.2

*** $p < 0.001$ vs. 3-BPA and 3-BPA-Fru by Tukey's multiple comparisons test.

Supplementary Table S8

Water solubility of phenylalanine, tyrosine, and *meta*-tyrosine

	Phenylalanine	Tyrosine	*meta*-Tyrosine
Solubility (g/L) (in water, 25°C, n = 3)	15.3 ± 0.1	0.34 ± 0.01	9.8 ± 0.1

Supplementary Methods
General materials and methods for synthesis

All chemical reagents and solvents were obtained from commercial suppliers and used without further purification. Thin layer chromatography (TLC) was performed on silica gel 60 F254 precoated aluminum sheets (Sigma-Aldrich Japan, Tokyo, Japan) and visualized by UV-light or $KMnO_4$ staining. Chromatographic purification was accomplished using a flash column chromatography system (Isolera Four, Biotage Japan Ltd., Tokyo, Japan) equipped with silica gel flash cartridges (Rening series, Biotage Japan). ^{1}H- and ^{13}C-NMR spectra were recorded in deuterated solvents on a Varian Mercury 400 (400 MHz) spectrometer or DD2 NMR Spectrometer (600 MHz, Agilent, CA, USA), calibrated to tetramethylsilane (= 0 ppm) or trimethylsilyl propanoic acid (for D_2O, = 0 ppm). ^{11}B-NMR spectra were recorded in D_2O on DD2 NMR Spectrometer (600 MHz), calibrated to boron trifluoride etherate (= 0 ppm). Multiplicities are abbreviated as follows: s = singlet, d = doublet, t = triplet, m = multiplet, dd = double doublet, dt = double triplet. Electrospray ionization mass spectrometry (ESI-MS) was carried out on LCMS-8045 (Shimadzu Corp., Kyoto, Japan).

Synthesis of compound **a**

3-Iodophenylboronic acid (2.0 g, 8.07 mmol, BLD pharm, Shanghai, China), *N*-Methyliminodiacetic Acid (MIDA, 3.56 g, 24.2 mmol, BLD pharm), and molecular sieves (2.88 g, Nacalai Tesque, Kyoto, Japan) were dissolved in dry DMF (75 mL). The reaction mixture was heated to 120°C. On the complete consumption of 3-Iodophenylboronic acid monitored by TLC, the reaction mixture was cooled to rt and concentrated under reduced pressure. The crude mixture was re-dissolved in acetone and unreacted MIDA was removed by filtration and the residue was purified by silica gel chromatography (Hexanes/EtOAc = 20/80 to 0/100) to afford **a** (2.69 g, 93%) as a white solid. ^{1}H NMR (400 MHz, DMSO-d_6) 7.74–7.69 (m, 2H), 7.40 (d, 1H), 7.12 (t, 1H), 4.28 (d, 2H), 4.08 (d, 2H), 2.46 (s, 3H).

Synthesis of compound **b**

Preparing *tert*-butyl N,N'-diisopropylcarbamimidate

To an oven-dried 100 mL flask charged with N,N'-Diisopropylcarbodiimide (10.0 mL, 64.6 mmol, Watanabe chemical industries, Ltd., Hiroshima, Japan) and *t*-BuOH (7.1 mL, 74.8 mmol, Nacalai Tesque) was added CuCl (64.0 mg, 0.65 mmol) under argon. The resulting mixture was stirred for 72 h at rt.

A solution of commercially available Boc-*L*-serine (5.25 g, 26.0 mmol, Watanabe chemical industries, Ltd.) in DCM (50 mL) was added to chilled *tert*-butyl N,N'-diisopropylcarbamimidate (12.9 g, 64.6 mmol; freshly prepared according to above). Reaction was stirred in an ice bath for 30 min, and then allowed to warm to rt and stir overnight. Hexanes (65 mL) was added to the reaction, and it was stirred for 15 min. The suspension was filtered through a pad of celite to remove the diisopropyl urea by-product, and the filtrate was concentrated under reduced pressure. The resulting residue was purified by silica gel chromatography (Hexanes/EtOAc = 80/20 to 50/50) to afford **b** (4.7 g, 69.4%) as a white solid. ^{1}H NMR (400 MHz, $CDCl_3$) 5.46–5.45 (m, 1H), 4.26 (br s, 1H), 3.91–3.90 (m, 2H), 1.49 (s, 9H), 1.46 (s, 9H).

Synthesis of compound **c**

Triphenylphosphine (3.21 g, 12.2 mmol, Wako Pure Chemical Industries, Osaka, Japan) and imidazole (0.83 g, 12.2 mmol, Nacalai Tesque) were dissolved in DCM (20 mL) in a round bottom flask. The mixture was stirred under argon in an ice bath. Iodine (3.10 g, 12.2 mmol) was added all-at-once. The mixture was then removed from the ice bath and allowed to warm to rt over 10 min. The reaction was cooled back down in an ice bath, and a solution of **b** (2.13 g, 8.15 mmol) dissolved in DCM (8 mL) was added dropwise under argon. The reaction was stirred at 0 °C for 30 min, then warmed to rt for 30 min. The reaction mixture was purified by silica gel chromatography (Hexanes/EtOAc = 100/0 to 90/10) to afford **c** (1.06 g, 35%) as a white solid. ^{1}H NMR (400 MHz, $CDCl_3$) 5.36–5.34 (m, 1H), 4.36–4.34 (m, 1H), 3.57–3.56 (m, 2H), 1.50 (s, 9H), 1.46 (s, 9H).

Synthesis of compound **d**

Zinc dust (600 mg, 9.0 mmol) was placed in an Ar-purged glass vial. Dry DMF (450 μL) and trimethylsilyl chloride (180.0 μL, 1.41 mmol, Tokyo Chemical Industry (TCI), Tokyo, Japan) were added to the vial, and the resulting mixture was stirred vigorously for 30 min at rt. The remaining solid was dried at 70 °C in reduced pressure. The activated zinc was then cooled to room temperature. A solution of **c** (558 mg, 1.50 mmol) in dry DMF (600 μL) was then added to the activated zinc. The reaction mixture was then stirred at rt. After completion of the insertion monitored by TLC, the zinc solution was allowed to settle using a centrifuge for 1 min at rt. The organozinc solution was removed via a microsyringe and added to a 10 mL flask containing $Pd(dba)_2$ (45 mg, 0.08 mmol, Sigma-Aldrich Japan), $P(2\text{-furyl})_3$ (27.9 mg, 0.12 mmol, TCI), and **a** (540 mg, 1.50 mmol), rinse with dry DMF (600 μL). After stirring for 3 h at rt, and resulting solution was directly purified by silica gel chromatography (Hexanes/EtOAc = 50/50 to 0/100) to afford **d** with unreacted MIDA boronate. Further purification was conducted by an RP-HPLC system equipped with a C18-reverse phase column (COSMOSIL 5C18-AR-II 10 ID × 250 mm; Nacalai Tesque) and detected by UV-absorption (Shimadzu). The mobile phase was a linear gradient of water and acetonitrile that increased from 40% to 80% acetonitrile over 20 min at a flow rate of 5.0 mL/min. The purified compound **d** (Retention time = 14.7 min) was lyophilized to obtain white powder (273 mg, 38%), and was characterized by ESI-MS. MS (ESI) *m/z* 477.2 $[M+H]^+$ (Chemical formula: $C_{23}H_{33}BN_2O_8$, calculated *m/z*: 476.2)

Synthesis of **3-BPA**

To a stirred solution of compound **d** in DCM (3 mL) was added trifluoroacetic acid (TFA, 3 mL, Nacalai Tesque) at rt. The reaction solution was allowed to stir for 2 h. TFA was removed and concentrated by evaporation under reduced pressure. To remove MIDA boronate, aqueous NaOH (1M) was added to the resulting residue and allowed to stir for 1 h at rt. Finally, aqueous HCl (1M) was added to the solution to neutralize, and the resulting aqueous solution was purified directly by RP-HPLC system equipped with a C18-reverse phase column (COSMOSIL 5C18-AR-II 10 ID × 250 mm; Nacalai Tesque, Kyoto, Japan). The mobile phase was the mixture of water and acetonitrile (95:5, v/v) at a flow rate of 5.0 mL/min. The purified **3-BPA** was lyophilized to obtain fluffy white crystal (98 mg, 82%). **3-BPA** was characterized by ESI-MS and ^{1}H and ^{13}C-NMR.

MS (ESI) *m/z* 210.2 $[M+H]^+$ (Chemical formula: $C_9H_{12}BNO_4$, calculated *m/z*: 209.1) ^{1}H NMR (600 MHz, D_2O): δ 7.68 (dt, 1H), 7.61 (br s, 1H), 7.44 (t, 1H), 7.41 (dt, 1H), 3.99 (dd, 1H), 3.28, (dd, 1H), 3.13 (dd, 1H). ^{13}C NMR (600 MHz, D_2O): δ 176.7, 137.4, 137.2, 135.8, 135.6, 134.7, 131.5, 58.9, 39.2.

Water solubility of *DL*-tyrosine, *DL-meta*-tyrosine, and *DL*-phenylalanine

DL-Tyrosine, *DL-meta*-tyrosine, and *DL*-phenylalanine were obtained from TCI. *DL*-tyrosine (5 mg), *DL-meta*-tyrosine (20 mg), or *DL*-phenylalanine (40 mg) was suspended in 1 mL of water and stirred at 25°C for 24 hr. After centrifugation, the supernatant was collected and dilution with water, followed by measuring UV-visible absorbance (*DL*-tyrosine; 275 nm, *DL-meta*-tyrosine; 278 nm, *DL*-phenylalanine; 257 nm) using spectrophotometer V-630 (JASCO Corp., Tokyo, JAPAN). The concentration was calculated from the calibration curve showing the relationship between the concentration and absorbance of each reagent.

Complex formation with fructose

3-BPA or 4-BPA and fructose were dissolved in choline buffer for in vitro experiment or 1N NaOH for in vivo experiment at a molar ratio of 1:2.6. The mixture was stirred until 3-BPA and 4-BPA had completely dissolved, and the pH was adjusted to 7.4 with 1N HCl. This solution was diluted with choline buffer for in vitro experiments and with PBS(-) for in vivo experiments. For ^{11}B-NMR measurements, 3-BPA and commercially available 4-BPA containing ^{11}B (Sigma Aldrich Japan) were used and prepared as above, and then diluted in PBS(-) prepared with D_2O.
